# The Influence of Reduced Susceptibility to Fluoroquinolones in *Salmonella enterica* Serovar Typhi on the Clinical Response to Ofloxacin Therapy

**DOI:** 10.1371/journal.pntd.0001163

**Published:** 2011-06-21

**Authors:** Christopher M. Parry, Ha Vinh, Nguyen Tran Chinh, John Wain, James I. Campbell, Tran Tinh Hien, Jeremy J. Farrar, Stephen Baker

**Affiliations:** 1 Wellcome Trust Major Overseas Programme, Oxford University Clinical Research Unit, The Hospital for Tropical Diseases, Ho Chi Minh City, Vietnam; 2 Centre for Tropical Diseases, University of Oxford, Oxford, United Kingdom; 3 Wellcome Trust Major Overseas Programme, Mahidol University-Oxford University Clinical Research Unit, Angkor Hospital for Children, Siem Reap, Cambodia; 4 The Hospital for Tropical Diseases, Ho Chi Minh City, Vietnam; 5 The Laboratory for Gastrointestinal Pathogens, Health Protection Agency, Colindale, United Kingdom; Massachusetts General Hospital, United States of America

## Abstract

**Background:**

Infection with *Salmonella enterica* serovar Typhi (*S.* Typhi) with reduced susceptibility to fluoroquinolones has been associated with fluoroquinolone treatment failure. We studied the relationship between ofloxacin treatment response and the ofloxacin minimum inhibitory concentration (MIC) of the infecting isolate. Individual patient data from seven randomised controlled trials of antimicrobial treatment in enteric fever conducted in Vietnam in which ofloxacin was used in at least one of the treatment arms was studied. Data from 540 patients randomised to ofloxacin treatment was analysed to identify an MIC of the infecting organism associated with treatment failure.

**Principal Findings:**

The proportion of patients failing ofloxacin treatment was significantly higher in patients infected with *S.* Typhi isolates with an MIC≥0.25 µg/mL compared with those infections with an MIC of ≤0.125 µg/mL (*p*<0.001). Treatment success was 96% when the ofloxacin MIC was ≤0.125 µg/mL, 73% when the MIC was between 0.25 and 0.50 µg/mL and 53% when the MIC was 1.00 µg/mL. This was despite a longer duration of treatment at a higher dosage in patients infected with isolates with an MIC≥0.25 µg/mL compared with those infections with an MIC of ≤0.125 µg/mL.

**Significance:**

There is a clear relationship between ofloxacin susceptibility and clinical outcome in ofloxacin treated patients with enteric fever. An ofloxacin MIC of ≥0.25 µg/mL, or the presence of nalidixic acid resistance, can be used to define *S.* Typhi infections in which the response to ofloxacin may be impaired.

## Author Summary

Typhoid fever is an infection of the bloodstream caused by the organism *Salmonella* Typhi (*S.* Typhi). Treatment with antimicrobials is critical for preventing severe infection and even death, yet antimicrobial resistant organisms have become a problem in many places where typhoid is common. Fluoroquinolones are a group of antimicrobials that are commonly used to treat typhoid, we analysed data from 540 enteric fever patients treated with ofloxacin (a fluoroquinolone) to identify a level of resistance (minimum inhibitory concentration (MIC)) from the infecting organism which is associated with treatment failure. The proportion of patients failing treatment was higher in those infected with a bacterium with an MIC≥0.25 µg/mL, compared with those infections with an MIC of ≤0.125 µg/mL. Treatment success was 96% when the ofloxacin MIC was ≤0.125 µg/mL, yet only 53% when the MIC was 1.00 µg/mL. Our data demonstrates that an *S.* Typhi bacterium with an ofloxacin MIC of ≥0.25 µg/mL correlates with a poor outcome when treated with this antimicrobial. Therefore, we propose an amendment in the current MIC guidelines for microbiology laboratories to aid clinicians treating typhoid and suggest the use of alternative therapy in these patients.

## Introduction

Enteric fever is a systemic infection caused by *Salmonella enterica* serovar Typhi (*S.* Typhi) and *Salmonella enterica* serovar Paratyphi A (*S.* Paratyphi A) [1]. Antimicrobial therapy is critical for the clinical management of enteric fever. The emergence and sustained circulation of antimicrobial resistant organisms have become problematic in many endemic regions [1], [2]. Multiple drug resistant *S.* Typhi (MDR: resistance to chloramphenicol, trimethoprim/sulfamethoxazole and ampicillin) have been widespread since the early 1990's and hinder effective treatment and limit alternatives.

Fluoroquinolones are commonly used for treating enteric fever and have been recommended by the WHO for the treatment of uncomplicated enteric fever caused by fully sensitive and MDR organisms [3]. Ciprofloxacin and ofloxacin were chosen for treating typhoid because of potent bactericidal activity against *S.* Typhi and *S.* Paratyphi A, *in vivo*, both drugs have plasma levels considerably in excess of the prevailing MICs and excellent intracellular penetration [4]. Widespread use of fluoroquinolone therapy for enteric fever has been followed by the emergence of *S.* Typhi and *S.* Paratyphi A isolates with elevated minimum inhibitory concentrations (MIC) to ciprofloxacin and ofloxacin across Asia and in parts of Africa [5], [6], [7], [8], [9]. These strains are associated with point mutations in the *gyrA* gene and occasionally the *parC* gene [5], [7], [9], [10], [11], [12]. To date, there have been few published reports of plasmid mediated quinolone resistance (PMQR) genes, such as *qnr*, *aac(6′)-Ib-cr*, or efflux pumps, in *S.* Typhi as described in some non-typhoidal *Salmonella* serovars [2], [13]. The identification of strains with reduced susceptibility to fluoroquinolones is important to guide treatment, yet these strains are categorised as susceptible by the current guidelines for fluoroquinolone disk susceptibility testing [14], [15], [16]. Such isolates can be identified by using resistance to nalidixic acid as a surrogate marker of fluoroquinolone susceptibility, although this is not completely reliable [17], [18]. Enteric fever caused by *S.* Typhi strains with an elevated MIC to ciprofloxacin and ofloxacin have been coupled with the failure of treatment with these antimicrobials and increased disease severity [12], [19], [20], [21], [22]. Yet, a clear correlation between the MIC of the infecting organism to fluoroquinolones and the clinical response to fluoroquinolone therapy has not been defined. The aim of this work was to study the relationship between the response to ofloxacin treatment and the ofloxacin MIC of the infecting isolate and define an MIC breakpoint that can predict a poor therapeutic response to ofloxacin. We have analysed individual patient data combined from seven open randomised controlled trials (RCTs) using a standard protocol that had been conducted in Vietnam of uncomplicated enteric fever caused by *S.* Typhi.

## Methods

### Ethics statement

The study was conducted according to the principles expressed in the Declaration of Helsinki and approved by the Institutional Review Board of the Hospital for Tropical Diseases and the additional hospital involved in the studies. All patients provided verbal informed consent (verbal informed consent was provided by the parents or guardian of children under 18 years of age) for the collection of samples and subsequent analysis.

### Study setting

We analysed the results of seven open RCTs for enteric fever conducted in southern Vietnam between 1992 and 2001 in which treatment with ofloxacin was used in at least one of the trial arms. All the RCTs were conducted using a standard protocol, except for the dose and duration of ofloxacin treatment and the alternative treatment regimens used. The RCTs were conducted at three study sites in southern Vietnam: The Hospital for Tropical Diseases, Ho Chi Minh City [23], [24], [25], [26]; Dong Thap Provincial Hospital, Cao Lanh, Dong Thap Province [27], [28] and Dong Nai Provincial Hospital, Bien Hoa, Dong Nai Province [29]. All studies were approved by the Ethical Committee of the Hospital conducting the study. The studies were conducted in accordance with ICH and Declaration of Helsinki guidelines.

### Clinical procedures

Patients with suspected uncomplicated enteric fever were allocated to one of each of the treatment groups in an open randomised comparison. A computer generated randomisation list was produced by a member of staff that was not otherwise involved in the study. The treatment allocations were kept in serially numbered sealed opaque envelopes that were only opened after the patient had been enrolled into the study. The treatment arms were ofloxacin (Oflocet, Hoescht Marion Roussel, Paris, France) at a dose that varied between 10 and 20 mg/kg/day orally in two-divided dose (maximum 400 mg twice daily) for between two and seven days (depending on the study, or the comparator). The comparator arms were, either, a different regimen of ofloxacin (in three RCTs) [24], [26], [28] ceftriaxone [25], cefixime [29], or azithromycin (in two RCTs) [23], [27].

Patients were excluded if; they refused consent, had evidence of progressive or complicated disease, had inability to swallow oral medication, had a history of significant underlying disease, had hypersensitivity to either of the trial drugs or were pregnant or lactating. Additionally, patients who gave a history of treatment with a fluoroquinolone, a third generation cephalosporin or a macrolide within one week of hospital admission were also excluded.

### Clinical definitions

In all seven studies, patients were examined daily until discharge from hospital, with particular reference to clinical symptoms and complications of the disease and body temperature was measured every six hours. Response to treatment was assessed by the resolution of clinical symptoms and signs, the fever clearance time (time from the start of treatment until the body temperature reached ≤38.0°C, and remained ≤38.0°C for 48 hours), the development of complications or death, any evidence of relapse of infection and persistent fecal carriage after the conclusion of treatment or at the one month follow up visit.

Clinical treatment failure was defined as the persistence of fever (>38°C) and other enteric fever related symptoms for more than seven days after the initiation of treatment or the development of severe complications (severe gastrointestinal bleeding, intestinal perforation, visible jaundice, myocarditis, pneumonia, renal failure, shock or an altered conscious level) during treatment which required a change in therapy. Microbiological treatment failure was defined as isolation of *S.* Typhi from blood or a sterile site after the completion of treatment. Post study fecal carriage was defined as a positive fecal culture, with an isolate having the same susceptibility pattern as the original isolate, after the end of the initial treatment and before hospital discharge.

Patients were requested to return for a follow up assessment at four weeks or earlier if their symptoms recurred. Clinical evidence of relapse was sought and one fecal culture was performed. A blood culture was performed if the symptoms and signs suggested relapse. A relapse was defined as a recurrence of symptoms and signs suggestive of enteric fever within the four week period after the patient had been discharged well from the hospital accompanied by a blood culture positive for *S.* Typhi. One month faecal carriage was defined as a positive faecal culture at the one month follow up visit, with an isolate having the same susceptibility pattern as the original isolate.

### Laboratory investigations and microbiology

A hematocrit, white cell, platelet count and blood differential count were performed with serum aspartate transaminase, alanine transaminase, creatinine levels and urinalysis before therapy was initiated. Aspartate transaminase and alanine transaminase measurements were repeated one day after the end of therapy. A full blood count was repeated if there was a suggestion of gastrointestinal bleeding or clinical evidence of anaemia. Blood and fecal cultures were obtained before therapy and in one study a bone marrow was also performed in selected patients [27]. A blood culture was performed on all patients a day after the end of treatment. In addition, three fecal specimens were cultured between two and four days after the end of treatment and at the one month follow up visit. Bacterial culturing was performed as previously described [23], [24], [25], [26], [27], [28], [29], colonies presumptive of *S.* Typhi were identified using standard biochemical tests and serotype-specific antisera (Murex Biotech, Dartford, England).

Antimicrobial sensitivities were performed by the modified Bauer-Kirby disc diffusion method with zone size interpretation based on CLSI guidelines [30]. Antimicrobial discs tested were chloramphenicol (30 µg), ampicillin (10 µg), trimethoprim -sulphamethoxazole (1.25/23.7 5 µg), ceftriaxone (30 µg), ofloxacin (5 µg), azithromycin (15 µg) and nalidixic acid (30 µg). Isolates were stored in protect beads (Prolabs, Oxford, United Kingdom) at −20°C for later ofloxacin MIC testing by agar plate dilution [31]. *Escherichia coli* ATCC25922 and *Staphylococcus aureus* ATCC25923 were used as control strains for these assays. An isolate was defined as MDR if it was resistant to chloramphenicol (≥32 µg/ml), ampicillin (≥32 µg/ml) and trimethoprim/sulfamethoxazole (≥8/152 µg/ml). An isolate was defined as nalidixic acid resistant if it had an MIC of ≥32 µg/ml [15].

### Statistical analysis

Analysis was restricted to patients in whom *S.* Typhi was isolated from blood or bone marrow culture prior to treatment with ofloxacin and in whom the ofloxacin MIC of the original infecting isolate had been determined. The pooled admission and outcome data for individual patients was compiled with respect to the ofloxacin MIC of the original infecting isolate. Proportions were compared with the Chi squared test, Fisher's exact test or analysis of variance. Normally distributed data were compared using the Student t-test, non-normally distributed data using the Mann Whitney U test or Kruskall Wallis test. The fever clearance time was compared using survival analysis and the log rank test. Independent risk factors for clinical failure in the clinical trials were determined by multivariate logistic regression, a *p* value of <0.05 was considered significant. Statistical analysis was performed using SPSS for Windows version 18 (SPSS Inc, Chicago, USA).

## Results

### Clinical features of enteric fever patients treated with ofloxacin

**Figure 1:**
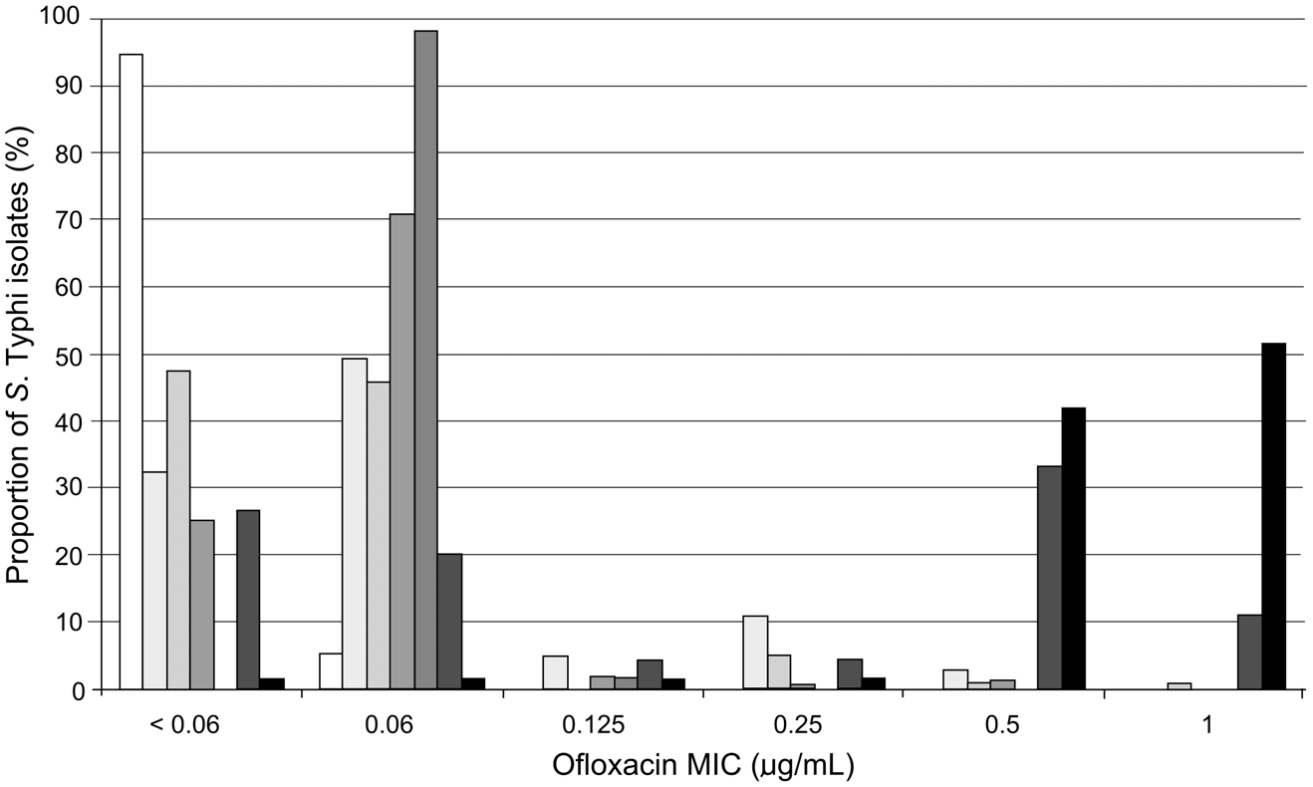
The distribution of *S.* Typhi MIC to ofloxacin in seven RCTs. Histogram showing the distribution of *S.* Typhi MICs (<0.06, 0.06, 0.125, 0.25, 0.5 and 1 µg/mL) to ofloxacin over seven individual randomised. The proportion of *S.* Typhi strains with the corresponding MIC are shaded accordingly, white; study TY1 (1992–1993) n = 19 [25], very light grey; study CT1 (1993–1994) n = 102 [26], light grey; study TY2 (1993–1996) n = 103 [24], mid grey; study DTC (1994–1995) n = 154 [28], dark grey; study DN (1995–1996) n = 55 [29], very dark grey; study TY3 (1997–1998) n = 45 [23] and black; study DTY2 (1998–2001) n = 62 [27].

A total of 540 patients infected with *S.* Typhi, treated with ofloxacin and in whom the ofloxacin MIC of the isolate was known from seven RCTs were available for analysis. The distribution of ofloxacin MIC values across the different trials is shown in Figure 1. The clinical features of the patients at the time of admission in relation to the ofloxacin MIC of the infecting *S.* Typhi isolate are shown in Table 1. There was heterogeneity among the six MIC (to ofloxacin) groups (≤.032 µg/ml, 0.064 µg/ml, 0.125 µg/ml, 0.25 µg/ml, 0.50 µg/ml and 1 µg/ml) with respect to a number of features, including the number of patients in each group. Abdominal pain, vomiting and positive fecal cultures were more frequently observed in patients infected with an isolate with a higher ofloxacin MIC. Nalidixic acid resistance was observed in none of the 423 *S.* Typhi isolates with an ofloxacin MIC of ≤0.06 µg/mL, in 2/12 (17%) of isolates with an MIC of 0.125 µg/mL and 104/105 (99%) of those with an MIC≥0.25 µg/mL (*p*<0.001) (Table 1).

**Table 1:** Admission features of 540 ofloxacin treated enteric fever patients recruited to clinical trials.

Admission features^a^	Ofloxacin MIC (µg/mL) of infecting isolate of *S.* Typhi						*p* b
	≤.032	0.064	0.125	0.25	0.50	1.0	
Number	152	271	12	20	47	38	
Duration of illness (days)	8 (7–12)	7 (6–10)	9 (6–11)	10 (7–12)	8 (6–14)	7 (4–10)	0.079
Abdominal pain [n (%)]	36 (24)	97 (36)	4 (33)	7 (35)	23 (49)	29 (76)	<0.001
Diarrhoea [n (%)]	86 (57)	175 (65)	10 (83)	12 (60)	38 (81)	31 (82)	0.005
Vomiting [n (%)]	26 (17)	51 (19)	1 (8)	4 (20)	12 (26)	20 (53)	<0.001
Hepatomegaly [n (%)]	76 (50)	195 (72)	8 (67)	12 (60)	27 (57)	24 (63)	0.001
Splenomegaly [n (%)]	22 (15)	44 (16)	2 (17)	6 (30)	2 (4)	3 (8)	0.081
Haematocrit (%)	35 (33–38)	34 (30–38)	32 (30–36)	36 (32-4)	34 (32–38)	35 (31–38)	0.042
White cell count	7.4 (5.7–9.2)	7.3 (5.8–9.2)	6.3 (5.3–8.4)	5.9 (5.0–9.0)	7.0 (5.4–9.5)	6.5 (5.3–8.2)	0.441
Platelet count	172 (152–203)	164 (140–200)	168 (156–243)	164 (149–185)	166 (128–227)	174 (123–225)	0.881
Fecal culture positive [n (%)]	16/132 (12)	23/239 (10)	1/9 (10)	4/11 (27)	13/40 (33)	8/32 (25)	0.001
Isolate MDR^c^ [n (%)]	121 (80)	218 (80)	10 (83)	19 (95)	45 (96)	45 (96)	0.248
Isolate NaR^d^ [n (%)]	0 (0)	0 (0)	2 (17)	20 (100)	46 (98)	38 (100)	<0.001

a ) continuous variables given as median (interquartile range), and proportions as number (%).

b ) Analysis of variance for proportions, Kruskall Wallis test for continuous variables.

c ) MDR – resistant to ampicillin, chloramphenicol and trimethoprim-sulfamethoxazole.

d ) NaR – resistant to nalidixic acid.

### Clinical response of enteric fever patients to ofloxacin therapy

Details of the duration and dosage of ofloxacin treatment and the corresponding response to therapy are shown in Table 2. The median duration of therapy for patients infected with *S.* Typhi isolates with an MIC<0.125 µg/mL was three days at a median dose of 11 mg/kg. In patients infected with *S.* Typhi isolates with an MIC of between 0.125–0.25 µg/mL the median duration of therapy was three days at a median dose of 13–15 mg/kg. For patients infected with isolates with an MIC>0.25 µg/mL the median duration of therapy was seven days at a dose of 16–18 mg/kg.

**Table 2:** Treatment response of 540 ofloxacin treated enteric fever patients recruited to clinical trials.

Treatment and outcome^a^	Ofloxacin MIC (µg/mL) of infecting isolate of *S.*Typhi						*P* b
	≤.032	0.064	0.125	0.25	0.50	1.0	
Number of patients	152	271	12	20	47	38	
Treatment duration (days)	3 (2–3)	3 (2–3)	3 (3–5)	3 (2–3)	7 (5–7)	7 (7-7)	<0.001
Mean ofloxacin dose/kg	11 (9–14)	11 (10–13)	13 (9–15)	15 (9–15)	16 (9–20)	18 (14–20)	<0.001
Mean ofloxacin dose/MIC	370 (317–489)	175 (167–222)	102 (75–120)	60 (36–60)	32 (17–40)	18 (14–20)	<0.001
Clinical failure [n (%)]	4 (3)	13 (5)	1 (8)	5 (25)	13 (28)	18 (47)	<0.001
Microbiological failure [n(%)]	1 (1)	2 (1)	0 (0)	1 (5)	1 (2)	2 (5)	0.499
Fever clearance time (days)	3.1 (2.5–4.2)	3.8 (2.8–4.8)	4.8 (3.1–7.4)	6.0 (3.5–10.3)	7.3 (5.3–9.8)	8.4 (6.8–11.3)	<0.001
Life-threatening complication n[%]	5 (3.3)	9 (3.3)	0 (0)	0 (0)	1 (2.1)	0 (0)	0.198
Post study feces positive [n (%)]	1/120 (1)	4/229 (2)	2/11 (18)	1/17 (6)	11/43 (26)	7/37 (20)	<0.001
1 month feces positive [n (%)]	0/61 (0)	0/83 (0)	1/4 (25)	1/19 (11)	3/29 (10)	1/29 (3)	0.001
Relapse [n (%)]	2/61 (3)	4/87 (5)	0/4 (0)	1/11 (9)	1/35 (3)	0/32 (0)	<0.001

a ) continuous variables given as medians (interquartile range), and proportions as numbers (%).

b ) Analysis of variance for proportions, Kruskall Wallis test for continuous variables and Log rank test for fever clearance time.

**Figure 2:**
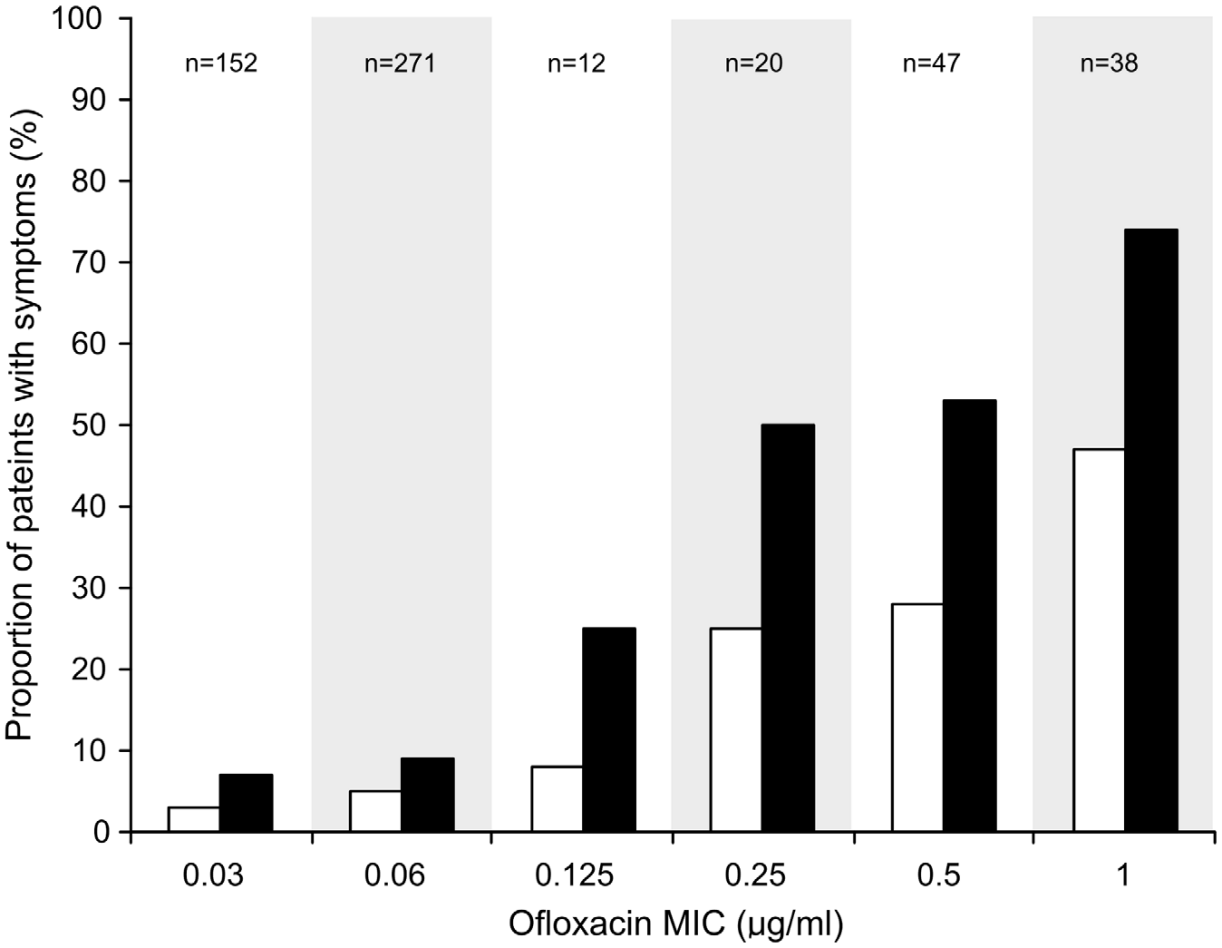
The relationship between increasing *S.* Typhi MIC to ofloxacin and clinical failure. Histogram showing the proportion of enteric fever patients who failed treatment (white columns) or had persistent fever (black columns) (>38°C) for more than seven days after the commencement of treatment. Data was combined from seven randomised clinical trials and is comprised from 540 children and adults recruited with uncomplicated enteric fever. The patients are divided according to the MIC to ofloxacin of the infecting isolate.

**Figure 3:**
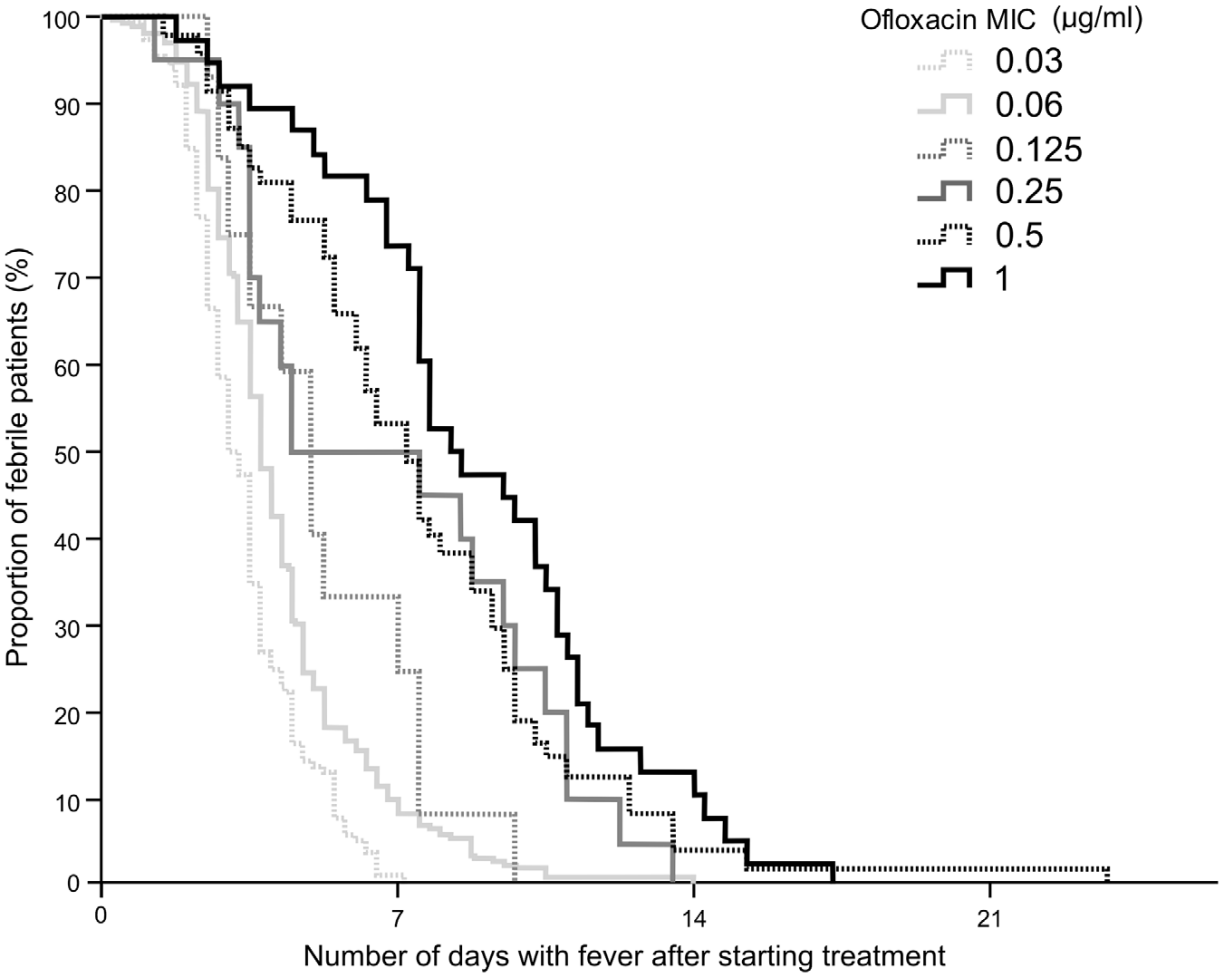
The duration of febrile episodes in enteric fever patients infected with *S.* Typhi organisms with a range of MICs to ofloxacin. Kaplan Meir curve showing the proportion of patients remaining febrile (>38°C) after the start of treatment with ofloxacin. Data is composed from fever clearance times of 540 children and adults with uncomplicated enteric fever recruited to seven randomised clinical trial and treated with oral ofloxacin. The curves are divided according to the ofloxacin MIC of the infecting isolates and are highlighted on the diagram.

We calculated the ratio of the administered dose of ofloxacin (mg/kg) to the MICs of the infecting isolates. The ratio of administered dose to bacterial MIC declined from 370 in patients infected with an *S.* Typhi isolate with an MIC of ≤0.03 µg/mL to 18 in those infected with an *S.* Typhi isolate with an MIC of 1.0 µg/mL. Despite a longer duration of treatment at a higher dosage, the proportion of patients failing treatment was significantly higher in the patients infected with *S.* Typhi isolates with an MIC≥0.25 µg/mL compared with those infected with isolates with an MIC of ≤0.125 µg/mL (*p*<0.001). Concurrently, the time to fever clearance was significantly longer in the patients with a higher MIC (Figure 2 and Table 2). There was an evident relationship between fever clearance time and the MIC of the infecting organism, as shown in Figure 3. Furthermore, the proportion of patients with a positive faecal culture, immediately post study treatment and at one month follow-up was also significantly greater among the patients infected with an isolate with a higher MIC to ofloxacin, *p*<0.001 and *p* = 0.001 respectively (Table 2).

Of the 540 patients, 15 (2.8%) developed a potentially life threatening complication during the course of treatment (gastrointestinal bleed requiring transfusion, hemodynamic shock, suspected myocarditis, encephalopathy, pneumonia) and one patient died of suspected myocarditis and shock. There was no significant difference in the rate of life-threatening complications for the patients infected with isolates with different ofloxacin MICs (Table 2).

### Predicting clinical failure to ofloxacin therapy in enteric fever patients

Univariate analysis was performed to identify the factors that were associated with clinical failure. The presence of abdominal pain (*p*<0.001), diarrhoea (*p* = 0.03), vomiting (*p*<0.001), lower hematocrit (*p* = 0.03), lower white cell count (*p* = 0.04), lower platelet count (*p* = 0.002), infection with an *S.* Typhi organism with a higher ofloxacin MIC (*p*<0.001), prolonged duration of ofloxacin treatment (*p*<0.001) and a higher administered dose of ofloxacin (*p* = 0.009) were all associated with clinical failure. Duration of therapy was deliberately increased as the MIC increased. If this factor is not included in the model, the independent variables associated with clinical failure with ofloxacin treatment using a multivariate logistic regression model were, abdominal pain, Odds Ratio (OR) 0.461, 95% confidence Interval (95% CI) 0.233–0.969, *p* = 0.033, vomiting; OR 0.427, 95% CI; 0.208–0.876, *p* = 0.020, hematocrit; OR 0.924, 95% CI; 0.861–0.992, *p* = 0.033, platelet count; OR 0.990, 95% CI; 0.984–1.00, *p* = 0.001) and the ofloxacin MIC of the infecting isolate; OR 17.08, 95% CI; 6.62–44.04, *p*<0.001.

To define an appropriate ofloxacin MIC breakpoint and the use of nalidixic acid resistance to define clinical failure to ofloxacin, the clinical success rate in each MIC group was calculated (Table 3). The data stratified by MIC to ofloxacin suggests a treatment success rate with ofloxacin given for a median duration of 3 days at 11–13 mg/kg of 96% in 435 patients infected with an isolate with an ofloxacin MIC of ≤0.125 µg/mL; 73% in 67 patients infected with isolate with an ofloxacin MIC between 0.25 and 0.5 µg/mL with treatment durations between 3 to 7 days at 15–16 mg/kg; and 53% in 38 patients infected with an isolate with an ofloxacin MIC of 1.00 µg/mL despite a median duration of treatment of 7 days at 18 mg/kg. The success rate in 434 patients infected with a nalidixic acid susceptible isolate was 96% compared with a 65% success rate in the 106 patients infected with a nalidixic acid resistant isolate.

**Table 3:** Ofloxacin MIC breakpoints for *S.* Typhi infection which predict ofloxacin treatment success.

Breakpoint value	Number successfully treated/total number treated (%)	Odds ratio (95% CI)
Ofloxacin MIC<0.06 µg/mLOfloxacin MIC≥0.06 µg/mL	148/152 (97.4%) 144338/388 (87.1%) 314	5.47 (1.95–21.20)
Ofloxacin MIC<0.12 µg/mLOfloxacin MIC≥0.12 µg/mL	406/423 (96.0%)80/117 (68.4%)	11.00 (5.71–21.88)
Ofloxacin MIC<0.25 µg/mLOfloxacin MIC≥0.25 µg/mL	417/435 (95.9%)69/105 (65.7%)	12.09 (6.24–23.81)
Ofloxacin MIC<0.50 µg/mLOfloxacin MIC≥0.50 µg/mL	432/455 (94.9%)54/85 (63.5%)	10.78 (5.6–20.77)
Ofloxacin MIC<1.00 µg/mLOfloxacin MIC≥1.00 µg/mL	466/502 (92.8%)20/38 (52.6%)	11.65 (5.26–25.36)
Nalidixic acid susceptibleNalidixic acid resistant	417/434 (96.1%)69/106 (65.1%)	13.15 (6.74–26.20)

## Discussion

In this study we used individual patient data from seven randomised controlled trials to characterise the relationship between ofloxacin susceptibility and outcome in ofloxacin treated patients with uncomplicated enteric fever. There was clear relationship between a higher ofloxacin MIC of the infecting isolate and a declining response to ofloxacin. Although the trials were conducted according to a standard protocol, the duration and dosage of ofloxacin treatment was not standard across all patient groups and the duration for some patients was shorter than would be routinely recommended. Despite this, a successful response in 96% of patients to an average duration of treatment of three days among infections with an ofloxacin MIC<0.25 µg/mL indicates the efficacy of ofloxacin when isolates are fully susceptible and justifies comparison with the response to infections where the isolate had a higher MIC and the ofloxacin dose used was higher and duration longer.

Our data suggests an MIC breakpoint for ofloxacin of ≥0.25 µg/mL or the presence of nalidixic acid resistance could be used to define infections in which the response to ofloxacin is impaired. The ciprofloxacin MIC is usually one dilution less than ofloxacin, and this implies a ciprofloxacin breakpoint of ≥0.125 µg/mL, a breakpoint already suggested [32], [33]. The small number of infections with isolates with an MIC of 0.125 µg/mL is a limitation and more information on the response to treatment at this MIC would be valuable. As nalidixic acid resistance is not a reliable proxy marker for reduced fluoroquinolone susceptibility in *S.* Typhi [17] we endorse the re-evaluation of disk susceptibility breakpoints for fluoroquinolones against *S.* Typhi by the clinical and laboratory standards institute (CSLI) [34]. This study only includes infections with *S.* Typhi so we can only propose applying similar breakpoints for *S.* Paratyphi A. Antimicrobial resistant *S.* Paratyphi A is an emerging problem in Asia and similar clinical characterisation of *S.* Paratyphi A infections is required [35].

A number of previous studies have reported an impaired response in typhoid fever to ciprofloxacin or ofloxacin when the isolates have decreased susceptibility. In general these studies are case reports or case series, mostly retrospective, or small numbers of patients in clinical trials. Despite these reports there has been a reluctance to change the MIC and disc susceptibility breakpoints for ciprofloxacin and ofloxacin and *Salmonella*. The data presented in this study were collected prospectively in the context of randomised controlled trials with uniform methods of collecting the treatment outcomes, during a period when the proportion of *S.* Typhi strains with reduced susceptibility to ciprofloxacin and ofloxacin changed from negligible to the majority. Reduced susceptibility to fluoroquinolones in the strains isolated during the period when these studies were conducted were predominantly associated with point mutations in *gyrA* and were typical of strains from other locations at this time [5]. This is a unique set of data that is unlikely to be repeated, with sufficient numbers of cases to provide a clear and definitive demonstration that an elevated MIC to ofloxacin is associated with treatment failure. We feel that the data are sufficiently convincing that it should now lead to a change in the MIC and disc susceptibility breakpoints for *S.* Typhi with ofloxacin and, potentially, ciprofloxacin.

Pharmacokinetic and pharmacodynamic (PK-PD) parameters are likely to be an important factor in the response to fluoroquinolone therapy. A study using an *in vitro* model of *S.* Typhi infection and Monte Carlo simulations to explore PK-PD parameters that were predictive of efficacy found that the free drug area under the concentration-time curve from 0 to 24 h/MIC ratio (AUC/MIC ratio) was the parameter most predictive of efficacy and that a ratio of 105 corresponded to 90% of maximal activity [36]. The pharmacokinetics of ofloxacin was not measured in the trials studied here. However, using the dose/kg of ofloxacin given as a crude measure of drug exposure, the data in this analysis suggests that when the ratio of the mean ofloxacin dose/kg divided by the MIC of the infecting organism is above 100 the treatment response is satisfactory, but when it is below 100 the treatment response is impaired. It should be noted that the plasma concentration of fluoroquinolone in individual patients given the same dose is variable. This, as well as other factors such as age and immune status, may explain why not all patients that have an organism with an MIC≥0.25 µg/mL will fail therapy. We suggest that future trials in enteric fever should include pharmacokinetic measurements to allow proper analysis of PK-PD parameters.

Some studies have suggested a higher rate of life-threatening complications in patients infected with *S.* Typhi isolates with decreased susceptibility to fluoroquinolones [22]. In this study there were no significant differences in the rate of life-threatening complications between the patients infected with isolates with different MICs. The rate of patients with life-threatening complications (2.2%) was lower than would be the case in unselected patients, as the subjects selected for these trials were specifically screened for uncomplicated disease at enrolment.

### Conclusions

*S.* Typhi strains that exhibit MICs to ofloxacin that are higher than those observed in this study are emerging and the ongoing use of fluoroquinolones, such as, ofloxacin and ciprofloxacin, to treat *S.* Typhi infections with reduced susceptibility may be driving their selection [13], [37], [38]. The increased fecal carriage following ofloxacin treatment of isolates with reduced ofloxacin susceptibility is also likely to drive increased local transmission. We suggest azithromycin, a third generation cephalosporin, or a later generation fluoroquinolone to be considered as alternatives for MDR *S.* Typhi isolates with an ofloxacin MIC of ≥0.25 µg/mL [20], [27], [39], [40]. Yet, there is an obvious risk of emerging resistance to these alternative antimicrobial agents [41] and dosing regimens need careful design to prevent this. Our data demonstrates that an *S.* Typhi isolate with an ofloxacin MIC of ≥0.25 µg/mL correlates with a poor clinical outcome when treated with this antimicrobial and we propose an amendment in the *S.* Typhi susceptibility breakpoints for ofloxacin and ciprofloxacin [34].
